# Inhibition of LPS-Induced Microglial Activation by the Ethyl Acetate Extract of *Pueraria mirifica*

**DOI:** 10.3390/ijerph191912920

**Published:** 2022-10-09

**Authors:** Nattinee Jantaratnotai, Anusorn Thampithak, Pongsak Utaisincharoen, Darawan Pinthong, Pimtip Sanvarinda

**Affiliations:** 1 Department of Pharmacology, Faculty of Science, Mahidol University, Bangkok 10400, Thailand; 2 Division of Pharmacology and Biopharmaceutical Sciences, Faculty of Pharmaceutical Sciences, Burapha University, Chonburi 20131, Thailand; 3 Department of Microbiology, Faculty of Science, Mahidol University, Bangkok 10400, Thailand

**Keywords:** *Pueraria mirifica*, microglia, inflammation, nitric oxide, cytokines

## Abstract

Microglial activation has been found to play a crucial role in various neurological disorders. Proinflammatory substances overproduced by activated microglia, such as cytokines, chemokines, reactive oxygen species, and nitric oxide (NO), can result in neuroinflammation that further exacerbates the course of the diseases. This study aimed to explore the anti-inflammatory effect of the ethyl acetate extract of *Pueraria mirifica* on microglial activation. Lipopolysaccharide (LPS)-induced inflammation was used as a model to investigate the effects of *P. mirifica* on HAPI (highly aggressive proliferating immortalized), a rat microglial cell line. Administration of ethyl acetate extract from the tuberous roots of *P. mirifica* to HAPI cells dose-dependently reduced NO production and iNOS expression induced by LPS. Attenuation of IRF-1 (interferon regulatory factor-1) induction, one of the transcription factors governing iNOS expression, suggested that the inhibitory effect on NO production by the plant extract was at least partially mediated through this transcription factor. In addition, LPS-stimulated mRNA expression of MCP-1 (monocyte chemoattractant protein-1), IL-6 (interleukin-6), and TNF-α (tumor necrosis factor-α) was also suppressed with *P. mirifica* extract pretreatment. This study indicates that the ethyl acetate extract of *P. mirifica* could potentially serve as an anti-inflammatory mediator and may be useful in relieving the severity of neurological diseases where microglia play a role.

## 1. Introduction

Microglia are the principal immune effector cells in the central nervous system (CNS), with an important role in the pathophysiology of diverse neurological diseases such as Alzheimer’s disease (AD), Parkinson’s disease (PD), multiple sclerosis (MS), AIDS dementia complex, and traumatic brain injury [1]. Activated microglia secrete a broad inflammatory repertoire including tumor necrosis factor-α (TNF-α), interleukin-6 (IL-6), IL-1β, IL-8, monocyte chemoattractant protein-1 (MCP-1), nitric oxide (NO), and reactive oxygen species (ROS) [2]. Overproduction of these substances has been shown to be neurotoxic and can deteriorate the courses of such diseases. Conversely, amelioration of microglial activation and subsequent reduction in these cytotoxic factors has proven beneficial for decreasing the severity of the diseases [3].

Naturally consumed products such as soybeans, clover, and oilseeds contain phytoestrogens, e.g., genistein and coumestrol, which are plant chemicals known to exert estrogen’s effects [4,5]. Data from considerable literature confirm the immunoregulatory role of phytoestrogens in various models [6,7]. The responsible mechanisms include the modulation of inducible NO synthase (iNOS), cyclooxygenase-2 (COX-2), and cytokine expression [8,9,10]. Regarding the neurological aspect, less information is known. One report on macelignan indicated that it potentially prevented lipopolysaccharide (LPS)-induced microglial activation of iNOS and COX-2 expression, as well as TNF-α and IL-6 production [11]. Genistein was able to diminish the induction of major histocompatibility complex (MHC) II, NO, and proinflammatory cytokines in microglia and astrocytes, but the mechanisms were not demonstrated [11,12,13]. So far, there have been no studies into the effects of other phytoestrogens such as daidzein on microglia.

This led us to study a phytoestrogenic plant found only in Thailand, *Pueraria mirifica* Airy Shaw and Suvatabandhu, which belongs to the family Leguminosae and the same subfamily as soy (Papilionoideae). It has been used as a rejuvenating folk medicine for over a century, as documented in “Treatise on the Drug made from the Tubers of the Kwao Vine by Luang Anusar Sunthorn, Special Commissioner, Chiengmai. May 1931” [14]. Active principles in the tuberous roots contain genistein, daidzein, kwakhurin, coumestrol, tuberosin, daidzin, puerarin, mirificin, puemiricarpene, miroestrol, and deoxymiroestrol [15,16].

Regarding CNS effects, two recent studies examined the effects of *P. mirifica* in ovariectomy- and orchidectomy-induced cognitive impairment in rats. Both found protective effects of the plants that could be due to its antioxidant and anti-inflammatory properties [17,18]. However, the mechanism of *P. mirifica* on microglia has not been explored before. We hypothesized that with abundant phytoestrogens present in *P. mirifica*, the plant would have a similar anti-inflammatory effect. Therefore, LPS, the most commonly used immunostimulator of microglia, was applied as a model in the present study. To investigate the inhibitory activity of ethyl acetate extract of *P. mirifica* in LPS-activated microglia, this study assessed NO/iNOS production and its upstream regulators interferon regulatory factor-1 (IRF-1) and interferons (IFNs), antioxidation, and cytokine expression. Studying *P. mirifica* could help us understand more about the roles of phytoestrogens in the CNS and their potential impact on diseases related to neuroinflammation.

## 2. Materials and Methods

### 2.1. Extraction of Pueraria mirifica

The tuberous roots of *P. mirifica* were collected in Chiangmai province, Thailand. The herbarium voucher specimen (BKF no. 126919) was identified and deposited at the Forest Herbarium, Royal Forest Department, Bangkok, Thailand. The plant materials were washed, dried, sliced into pieces, and ground to powder. Two grams of plant powder were subsequently extracted with 250 mL of ethyl acetate for 4 h in reflux apparatus at room temperature. The eluted solution was evaporated to dryness with a rotary evaporator. The brownish residue was collected and dissolved in dimethyl sulfoxide for the experiment.

### 2.2. Cell Cultures and Treatments

Highly aggressive proliferating immortalized (HAPI) microglial cells isolated from rat brains were characterized by Dr. Poonlarp Cheepsunthorn et al. [19], and were generously provided by Dr. James R. Connor (Department of Neuroscience and Anatomy, Hershey Medical Center, Pennsylvania State University, College of Medicine, Hershey, PA, USA). They were maintained in Dulbecco’s modified Eagle’s medium (DMEM; Life Technologies, Grand Island, NY, USA) supplemented with 5% heat-inactivated fetal bovine serum (FBS; Life Technologies) at 37 °C under a humidified 5% CO_2_ and 95% air atmosphere. In all experiments, cells were left to acclimate for 24 h before any treatments. *P. mirifica* was always added 1 h prior to LPS (from *Escherichia coli* 026:B6, Sigma, St. Louis, MO, USA) or sodium nitroprusside (SNP; Sigma).

### 2.3. Assessment of Cell Viability

The number of viable cells was determined by the mitochondrial conversion of yellow MTT (3(4,5-dimethylthiazol-2-yl)-2,5-diphenyltetrazolium bromide) to purple formazan dye. MTT (Sigma) was dissolved in Hank’s balanced salt solution (HBSS; Sigma), filtered, protected from light, and stored at 4 °C. HAPI cells were seeded on 96-well plates at a density of 15,000 cells per well. The next day, cells were treated with various concentrations of *P. mirifica* extract or LPS (100 ng/mL) and incubated for 24 h. The medium was removed and 10 μL of 10 mg/mL MTT in HBSS was added to each well. The cultures were incubated for 4 h in a humidified atmosphere at 37 °C and 5% CO_2_. Then, MTT was removed, cells were solubilized with 100 μL dimethyl sulfoxide, and the absorbance was measured at 570 nm on a microplate reader (Wallac 1420 Victor plate reader, Perkin-Elmer, Foster City, CA, USA). The results are shown as the percentage of the control (no treatment).

### 2.4. Assessment of NO Production

HAPI cells were plated at 100,000 cells/well in 24-well plates in 5% FBS-containing DMEM and incubated with LPS (100 ng/mL) or SNP (100 μM) for 24 h. NO production was determined from the quantification of nitrite, its stable end product. At the end of the treatment, 100 μL of cell culture supernatant from each sample was placed in a 96-well plate and an equal volume of Griess reagent (Sigma) was added. After 15 min of incubation at room temperature, the optical density was read at 545 nm with an automated microplate reader (Wallac 1420). Sodium nitrite, diluted in culture media at a 0–100 μM concentration, was used as the standard curve.

### 2.5. Assessment of mRNA Expression

HAPI cells were plated on six-well plates at 300,000 cells/well in 5% FBS containing DMEM. Cells were harvested after 6 h of incubation with LPS and the total RNA was isolated using a NucleoSpin RNA kit (Macherey-Nagel, Dueren, Germany) according to the manufacturer’s protocol. The yield and purity of the total RNA were determined using a UV spectrophotometer at 260 and 280 nm. A volume of 1 μg total RNA from each sample was subjected to reverse transcription using a Superscript First-Strand Synthesis System (Life Technologies). PCR amplification was performed in a reaction volume of 30 μL, containing 2 μL of cDNA product and HotStarTaq™ DNA polymerase (Qiagen, Hilden, Germany), using a PTC-200 Peltier Thermal Cycler (MJ Research, Waltham, MA, USA). The temperature cycling conditions of amplification were as follows: 15 min at 94 °C; 30 cycles of 94 °C for 40 s, 55 °C for 40 s, and 72 °C for 60 s; and a final extension at 72 °C for 10 min. Amplification of the constitutively expressed enzyme D-glyceraldehyde-3-phosphate dehydrogenase (GAPDH) was used as an internal control to assess the reverse transcription efficiency. The oligonucleotide primers used were: 5′-GCA GAA TGT GAC CAT CAT GG-3′(sense) and 5′-ACA ACC TTG GTG TTG AAG GC-3′(antisense) for iNOS; 5′-TTT GAG TGG AGG TTG GGA AG-3′ (sense) and 5′-GGT AGG GTT GGT TGG GTT TT-3′ (antisense) for IL-6; 5′-TCT ACA GAA GTG CTT GAG GTG GTT G-3′ (sense) and 5′-CCT GTT GTT CAC AGT TGC TGC C-3′ (antisense) for MCP-1; 5′-CTC CAG CTC CAA GAA AGG ACG-3′ (sense) and 5′-GAA GTT TCT GGT AAG TCT TCG-3′ (antisense) for IFN-β; 5′-AAC GCT ACA CAC TGC ATC TTG G-3′ (sense) and 5′-GAC TTC AAA GAG TCT GAG G-3′ (antisense) for IFN-γ; 5′-GTA GCC CAC GTC GTA GCA AA-3′ (sense) and 5′-CCC TTC TCC AGC TGG GAG AC-3′ (antisense) for TNF-α; and 5′-TCC CTC AAG ATT GTC AGC AA-3′(sense) and 5′-AGA TCC ACA ACG GAT ACA TT-3′(antisense) for GAPDH. The resulting amplification products were electrophoresed on 1.5% agarose gel, visualized by ethidium bromide staining, and photographed. Gel images were scanned using an image analysis system (Gel Doc 1000; Bio-Rad, Hercules, CA, USA). The intensities of specific PCR bands were quantitated in relation to GAPDH bands amplified from the same cDNA using Gene Tools analysis software (Syngene, Cambridge, UK).

### 2.6. Western Blot

HAPI cells (500,000 cells/well) were treated with 100 ng/mL of *P. mirifica* extract, with or without 100 ng/mL of LPS, for 6 h. Then cells were harvested and lysed with lysis buffer containing 62.5 mM Tris pH 6.8, 2% sodium dodecyl sulfate (SDS), 10% glycerol, and 5% 2-mercaptoethanol, followed by sonication on ice for 30 s. The protein lysates separated using 8% SDS-polyacrylamide gel electrophoresis were then transferred to nitrocellulose membrane (Schleicher & Schuell, Dassel, Germany). The membrane was blocked with 5% skim milk in PBS for 1 h before incubation (1:1000 dilution in blocking solution) with primary rabbit polyclonal antibody to mouse iNOS or IRF-1 (Santa Cruz Biotechnology, Santa Cruz, CA, USA) at 4 °C overnight. Horseradish peroxidase-conjugated swine anti-rabbit secondary antibody (Dako, Glostrup, Denmark; 1:1000 dilution in phosphate-buffered saline) was then applied, and the blots were developed using an enhanced chemiluminescence kit according to the protocol recommended by the manufacturer (Roche Diagnostic, Mannheim, Germany).

### 2.7. Statistical Analysis

Data are presented as the mean ± SD from three or more independent experiments. Statistical comparison between different treatments was carried out by one-way ANOVA with Tukey’s multiple comparison post hoc testing using SPSS program version 11. Differences were considered significant at *p* < 0.05.

## 3. Results

### 3.1. P. mirifica Extract Has No Effect on Cell Viability

**Figure 1:**
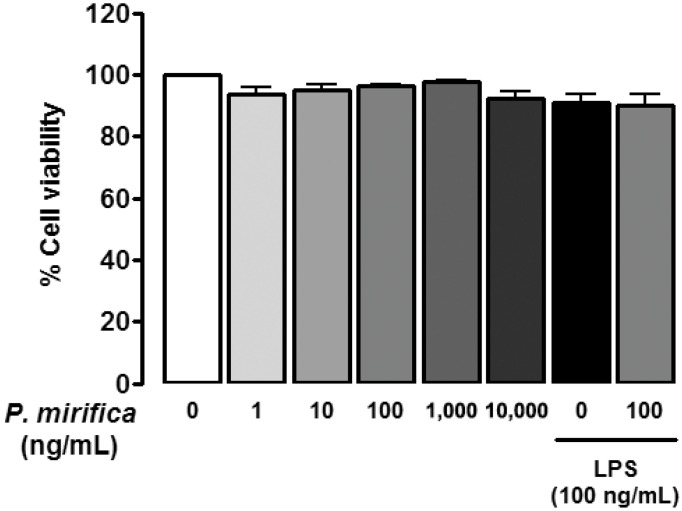
Effects of *P. mirifica* extract and LPS on microglial viability. HAPI cells (5 × 10^5^ cells/well) were treated with graded concentrations of ethyl acetate extract of *P. mirifica*, LPS (100 ng/mL), or coincubation of *P. mirifica* extract with LPS (both at 100 ng/mL) for 24 h and cell viability was determined with an MTT assay. Each value is the mean percentage ± SD when compared with the vehicle-treated control (100%) from three individual experiments.

The potential cytotoxicity of all chemicals administered was determined. HAPI cells were plated onto 96-well plates at 1.5 × 10^4^ cells/well and treated with graded concentrations of *P. mirifica* extract (10^−9^ to 10^−5^ g/mL) or LPS (100 ng/mL) in the absence or presence of *P. mirifica* extract (100 ng/mL). After 24 h, cultures were subject to MTT assay according to the protocol. Figure 1 shows that administration of either LPS or *P. mirifica* extract at any concentration did not significantly interfere with the viability of cells. In addition to this, co-treatment of *P. mirifica* extract with LPS did not further modulate cell numbers compared to cells treated with LPS alone. These results demonstrated that *P. mirifica* had no effect on microglial cell survival so these concentrations were chosen for subsequent experiments.

### 3.2. P. mirifica Extract Diminishes NO Production

**Figure 2:**
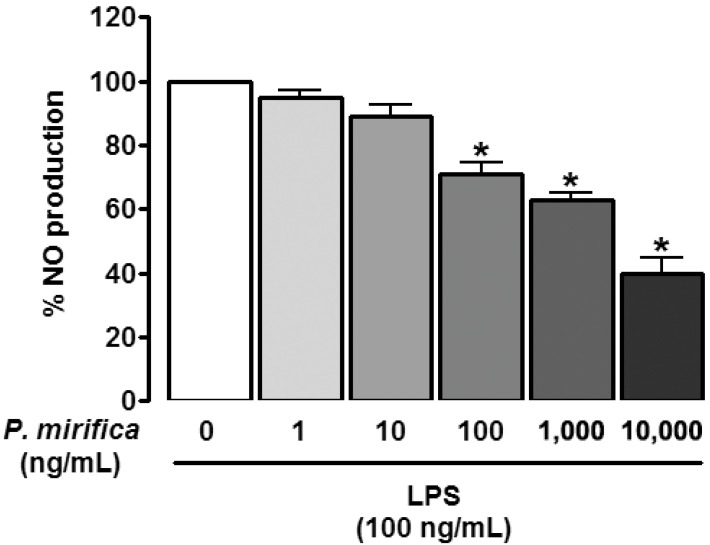
Effects of *P. mirifica* extract on LPS-induced NO production. HAPI cells (10^5^ cells/well) were treated with different concentrations of *P. mirifica* ethyl acetate extract for 1 h prior to administration of LPS (100 ng/mL) for 24 h. The amount of NO produced in the culture supernatants was determined with a Griess assay. Values are the mean percentage ± SD of six samples from three different experiments compared with the corresponding LPS-treated control (100%). * *p* < 0.05 compared with the LPS-treated control.

**Figure 3:**
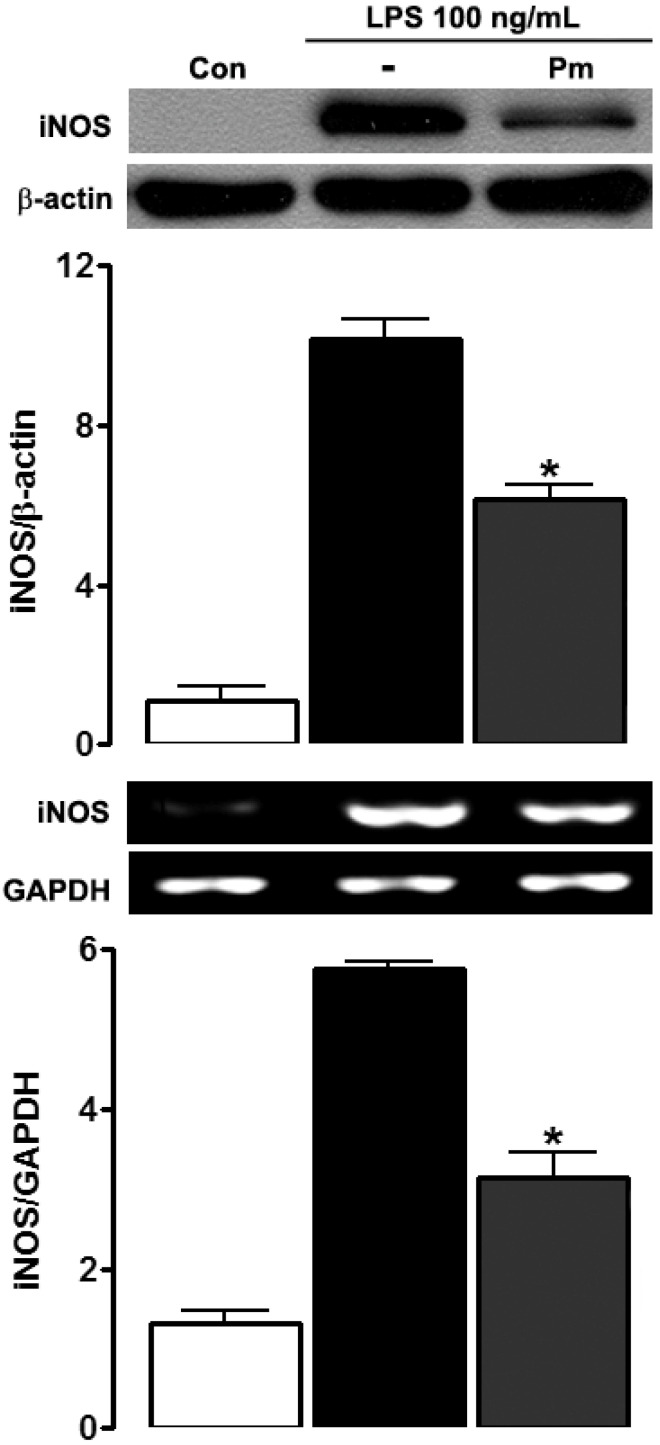
Effects of *P. mirifica* extract on LPS-induced iNOS protein and mRNA expression. HAPI cells (5 × 10^5^ cells/well) were pretreated with the ethyl acetate extract of *P. mirifica* (100 ng/mL) for 1 h followed by stimulation with LPS (100 ng/mL) for 6 h. The expression of iNOS protein was evaluated by Western blot analysis (upper panel), and iNOS mRNA (lower panel) was determined with semiquantitative RT-PCR. GAPDH was used as a reference for equal loading. The graphs illustrate levels of expression quantitated using a densitometer; for mRNA, the relative expression levels of iNOS transcript were normalized with respect to GAPDH. Data are representative of three separate experiments. Values are the mean ± SD. * *p* < 0.05 compared with the LPS-treated group.

We examined whether *P. mirifica* possessed any anti-inflammatory effect as determined by attenuation of NO production induced by LPS. Under normal conditions, HAPI cells barely produced detectable levels of NO. The exposure of HAPI cells to 100 ng/mL LPS for 24 h resulted in conspicuous NO production (20.02 ± 0.46 μM), while pretreatment for 1 h with *P. mirifica* extract diminished LPS-induced NO production in a dose-dependent manner (Figure 2). To ascertain if the inhibitory effect of *P. mirifica* extract on NO production was due to its direct antioxidant property, SNP, which is a NO donor, was administered at a concentration of 100 μM for 24 h following 1 h incubation with *P. mirifica* extract (100 ng/mL). Incubation of HAPI cells with SNP significantly resulted in increased NO levels; however, pretreatment with *P. mirifica* extract was unable to scavenge NO released by SNP, suggesting that *P. mirifica* extract did not possess an antioxidant effect at the concentrations used and that it could inhibit NO through another mechanism. The iNOS protein and mRNA expression as demonstrated by Western blot analysis and semiquantitative RT-PCR, respectively, were then assessed to see if *P. mirifica* exerted its effect through the modulation of iNOS. As presented in Figure 3, both iNOS protein and mRNA expression were downregulated in *P. mirifica*-pretreated cells as compared to marked upregulation of those treated with LPS alone. The graphs illustrate the quantification of the averaged densitometric data (*n* = 3). These results suggested the role of *P. mirifica* in the NO pathway at the transcriptional level of the iNOS gene or at the regulators upstream of it.

### 3.3. P. mirifica Extract Suppresses iNOS Expression via IRF-1 Inhibition

**Figure 4:**
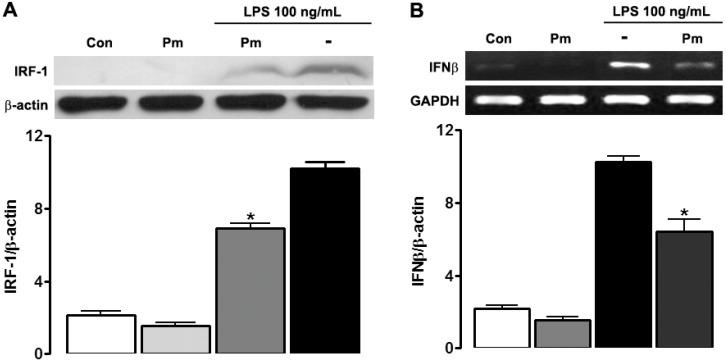
Effects of *P. mirifica* extract on LPS-induced IRF-1 protein and IFN-β mRNA expression. (**A**) HAPI cells (5 × 10^5^ cells/well) were pretreated with the ethyl acetate extract of *P. mirifica* (100 ng/mL) for 1 h followed by stimulation with LPS (100 ng/mL) for 6 h. The expression of IRF-1 protein was evaluated by Western blot analysis. The graphs illustrate levels of expression quantitated using a densitometer. Data are representative of three separate experiments. (**B**) HAPI cells (5 × 10^5^ cells/well) were treated with 100 ng/mL *P. mirifica* extract before LPS stimulation for 1 h. Total RNA was harvested and subjected to semiquantitative RT-PCR. GAPDH was used as a reference for equal loading. The relative expression levels of IFN-β transcript were normalized with respect to GAPDH. Results are representative of three independent experiments. * *p* < 0.05 compared with the LPS-treated group.

IRF is an important transcription factor for iNOS gene expression. We investigated whether IRF-1 protein expression was regulated by *P. mirifica* extract or LPS. Under normal condition, IRF-1 protein was slightly expressed in HAPI cells. Incubation with LPS (100 ng/mL) for 6 h stimulated IRF-1 expression and pre-exposure to *P. mirifica* extract for 1 h blocked this enhancement (Figure 4), indicating that LPS at least partially acted through IRF-1 to induce iNOS expression and finally NO production. As IFNs are known to regulate IRF-1 expression, the expressions of IFNs mRNA were detected. HAPI cells were treated with LPS (100 ng/mL) or pretreated with *P. mirifica* extract at the same concentration for 2 h. As demonstrated in Figure 4, LPS administration resulted in IFN-β upregulation while *P. mirifica* pretreatment could suppress it. On the other hand, IFN-γ was not expressed either in control or with LPS incubation suggesting that LPS had no effect on IFN-γ mRNA expression in HAPI cells.

### 3.4. P. mirifica Extract Inhibits Proinflammatory Cytokines Expression

**Figure 5:**
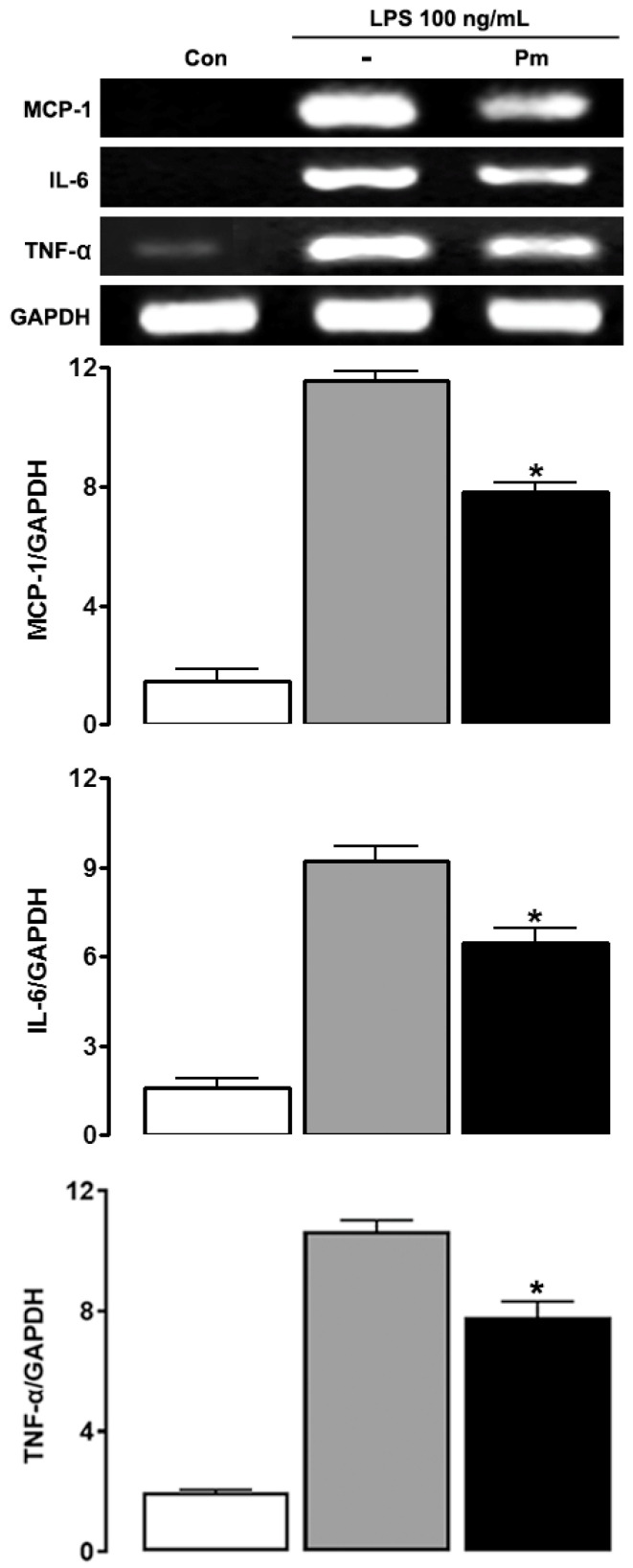
Effects of *P. mirifica* extract on LPS-induced MCP-1, IL-6, and TNF-α mRNA expression. HAPI cells (5 × 10^5^ cells/well) were pretreated for 1 h with 100 ng/mL *P. mirifica* extract before being stimulated with 100 ng/mL LPS for 6 h. Total RNA was harvested and subjected to semiquantitative RT-PCR. GAPDH was used as a reference for equal loading. The relative expression levels of MCP-1, IL-6, and TNF-α transcript were normalized with respect to GAPDH. The results are representative of three independent experiments. * *p* < 0.05 compared with the LPS-treated group.

To investigate whether the effect of *P. mirifica* extract was limited to NO or extended to other substances as well, the mRNA expression of MCP-1, a chemokine, and IL-6 and TNF-α, cytokines that are generally upregulated in reactive microgliosis, were examined with RT-PCR. Neither was constitutively expressed under physiological conditions, nor were they detected in cells treated with *P. mirifica* extract alone. However, when LPS (100 ng/mL) was treated for 6 h, they were strongly induced (Figure 5). Reductions in MCP-1 and IL-6 mRNA expression were observed when HAPI cells received 1 h of pretreatment with *P. mirifica* extract, suggesting that the plant may have a general anti-inflammatory effect involving not only the NO production pathway but also other pathways. Another possibility is that the extract might simply interfere early in the LPS signaling pathway.

## 4. Discussion

The present study demonstrates that the ethyl acetate extract from the tuberous roots of a Thai medicinal herb, *P. mirifica*, exhibits anti-inflammatory effects on reactive microglia. LPS was used to immunostimulate HAPI, a rat microglial cell line, to produce NO and enhance iNOS, IRF-1, IFN-β, MCP-1, TNF-α, and IL-6 expression. Pretreatment with *P. mirifica* ethyl acetate extract prominently suppressed these proinflammatory mediators’ expression by microglia, and these effects were not due to its antioxidant property since *P. mirifica* extract failed to diminish the NO production induced by SNP administration. Moreover, the administration of *P. mirifica* extract at all concentrations had neither a proliferative nor a cytotoxic effect on HAPI cells, suggesting a good safety profile. However, further studies are needed to confirm the systemic safety of the compound in vivo and in clinical trials.

Microglia are the prominent cells in the CNS that produce NO, which is one of the most studied substances generally used to reflect the degree of inflammation [1,2]. The involvement of excessive production of NO in several neurological disorders is well-established, and accumulating data have pointed out that inhibition of NO production provides significant neuroprotection [20]. In the current study, the administration of *P. mirifica* extract significantly suppressed NO produced from LPS-stimulated microglia without causing cytotoxicity to HAPI cells (Figure 1). A previous study found that the ethyl acetate–ethanol extract of *P. mirifica* possessed antioxidant activity against glutamate-induced neuronal injury [21]. However, in our study, *P. mirifica* extract failed to scavenge NO released from SNP, a direct NO donor. This could be because of the different models applied, the different extraction method of using ethyl acetate in the current study versus ethyl acetate–ethanol in the previous study, or the lower concentrations of *P. mirifica* extract (100 ng/mL vs. 10 µg/mL). These results implied that other mechanisms were involved.

The production of NO in microglia is mediated by iNOS [2]. The results of this study clearly demonstrated that LPS activation resulted in iNOS mRNA and protein upregulation, and pretreatment with *P. mirifica* extract blocked it. The synthesis of NO by iNOS is generally regulated at the transcriptional level through activation of NF-кB and IRF-1. Both transcription factors have a binding site on the iNOS promoter gene [22]. In the current study, we explored the less-studied pathway of IRF-1 induction instead of the more-studied NF-кB pathway. LPS administration leads to marked IFN-β production, which subsequently activates IRF-1 in an autocrine manner that later induces NO release in macrophages and microglia [23,24]. Moreover, NO production is blocked in the presence of the IFN-β neutralizing antibody, confirming the requirement of IFN-β in the pathway [24]. In the current study, we found that LPS stimulated IFN-β as well as IRF-1 expression, as expected, to induce NO production. Administration of *P. mirifica* extract blocked the expression of both IRF-1 and IFN-β. These results suggest that the extract was able to block this IFN-β/IRF-1/iNOS pathway. We did not find LPS to induce IFN-γ expression. This could be because IFN-γ is generally produced by NK cells and T cells [25]. Previous studies have found mouse macrophages to produce IFN-γ but not the macrophage cell line [26]. There could be a difference between the in vivo setting versus a cell culture in the cell-line setting [27]. 

In addition, the expression of MCP-1, TNF-α, and IL-6 chemokines and cytokines, which represent the inflammatory responses of microglial cells, was determined. Pretreatment with *P. mirifica* extract significantly attenuated the upregulated expression induced by LPS. The ethyl acetate extract of *P. mirifica* contains a host of phytoestrogenic chemicals, especially the highly estrogenic compounds miroestrol and deoxymiroestrol, which could be extracted by ethyl acetate and not in ethanol or aqueous solutions [15,28], so we chose this extract in the current experiment. These compounds are believed to be responsible for the actions of *P. mirifica* demonstrated in this study. Further studies are needed to determine which are the responsible compounds acting as anti-inflammatory agents; however, it is highly likely that many of the compounds, not just a single compound, act together to exert the plant’s various effects. This makes it beneficial to use the plant in a natural way rather than extracting just one active compound. In the future, studies that examine other stimulating agents (apart from LPS) will help provide more insight into the mechanism and the benefits of the plant extract.

## 5. Conclusions

This is the first study demonstrating the anti-inflammatory effects of *P. mirifica* ethyl acetate extract in LPS-activated microglial cells, as shown by the inhibitory effect on NO production, iNOS expression, and the regulator of iNOS expression (IFN-β and IRF-1), as well as the effect on MCP-1, TNF-α, and IL-6, without antioxidant or cytotoxic effects under the condition explored. This helps confirm the neuroprotective effect of *P. mirifica*, which may be used to suppress the activity of reactive microglia in various neurological disorders.

## Data Availability

The data presented in this study are available on request from the corresponding author.
